# Addressing the Ethnicity Gap in Catechol O-Methyl Transferase Inhibitor Trials in Parkinson’s Disease: A Review of Available Global Data

**DOI:** 10.3390/jpm14090939

**Published:** 2024-09-03

**Authors:** Karolina Poplawska-Domaszewicz, Naomi Limbachiya, Mubasher Qamar, Lucia Batzu, Shelley Jones, Anna Sauerbier, Silvia Rota, Yue Hui Lau, K. Ray Chaudhuri

**Affiliations:** 1 Department of Neurology, Poznan University of Medical Sciences, 60-355 Poznan, Poland; 2 Basic and Clinical Neuroscience Department, The Maurice Wohl Clinical Neuroscience Institute, Institute of Psychiatry, Psychology and Neuroscience, King’s College London, 5 Cutcombe Road, London SE5 9RX, UK; 3 Parkinson’s Foundation Centre of Excellence, King’s College Hospital, Denmark Hill, London SE5 9RS, UK; 4 Department of Neurology, University Hospital Cologne, Faculty of Medicine, 50937 Cologne, Germany; 5 Division of Neurology, Medical Department, Tengku Ampuan Rahimah Hospital, Klang 41200, Malaysia; andrealau38@gmail.com

**Keywords:** Parkinson’s disease, COMT, diversity, polymorphism, pharmacogenetics

## Abstract

Catechol-O-methyltransferase inhibitors (COMT-Is) have significantly improved the quality of life and symptom management for those at advanced stages of Parkinson’s Disease (PD). Given that PD is one of the fastest-growing neurodegenerative diseases worldwide, there is a need to establish a clear framework for the systematic distribution of COMT-Is, considering inter-individual and intra-individual variations in patient response. One major barrier to this is the underrepresentation of ethnic minority participants in clinical trials investigating COMT-Is. To investigate this, we performed a narrative review. We searched PubMed for clinical trials investigating COMT-Is in patients with PD and examined the ethnic diversity of cohorts. A total of 63 articles were identified, with 34 trials found to match our inclusion criteria. Among the 34 trials meeting our inclusion criteria, only 8 reported participants’ ethnic backgrounds. Our findings reveal a consistent underrepresentation of ethnic minority groups in trials investigating COMT-Is in PD cohorts—a trend that reflects broader concerns across clinical research. In this review, we explore potential reasons for the underrepresentation of ethnic minorities in clinical trials and propose strategies to address this issue.

## 1. Introduction

Parkinson’s disease (PD) is the second most common age-related neurodegenerative disease, predicted to afflict approximately 12.9 million people by 2040 [1]. The rising incidence is frequently attributed to longer life expectancy worldwide; however, the burden of PD is heterogeneous, disproportionately affecting low- and middle-income regions, including India and China [2]. Currently, Levodopa (LD) remains the most effective symptomatic treatment for PD. However, long term LD use is associated with motor and non-motor complications, collectively known as the “wearing-off” phenomenon, known to impair patients’ quality of life and increases caregiver burden [3,4]. A common first-line approach in response to this is the prescription of catechol O-methyl transferase inhibitors (COMT-Is) [3]. These inhibitors work by blocking the peripheral metabolism of LD by the COMT enzyme, thereby increasing the efficiency of LD and managing “off” periods. Entacapone and the newer third-generation COMT inhibitor, Opicapone, are widely used in clinical practice, whereas Tolcapone is generally not recommended due to its hepatotoxic side effects [5,6].

**Figure 1:**
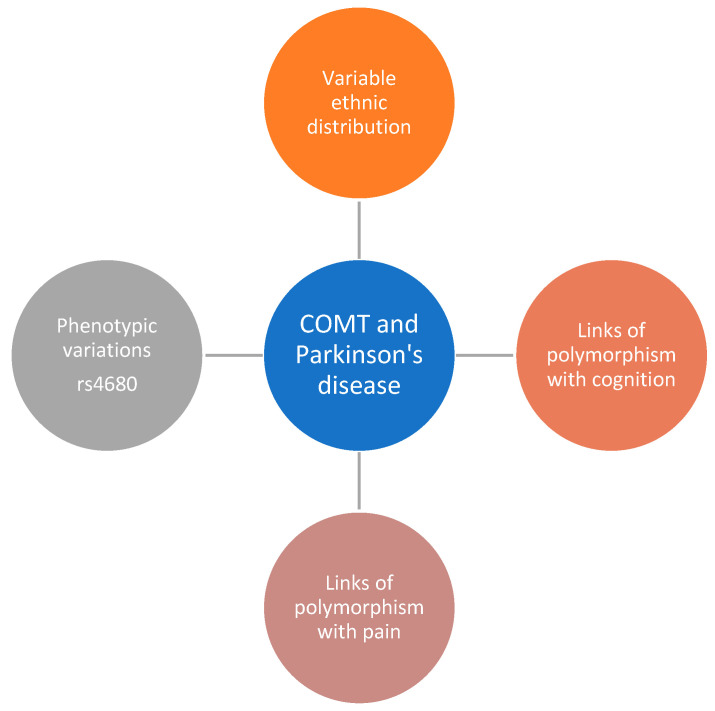
A summary and schematic representation of the various roles of Catechol O-Methyl Transferase and its functional consequences in Parkinson’s disease.

In global PD management, COMT-Is have strong potential to reduce the disease’s disproportionate burden. However, ensuring equitable use requires a distribution plan that addresses response variability, including drug interactions and genotype differences. (Figure 1). Support for this is demonstrated by studies that investigate COMT-Is among carriers of Val158Met (rs4680) in exon 4 This results in a three-tiered distribution of enzyme activity: high activity (COMT “GG” (Val/Val)), intermediate activity (COMT “GA” (Val/Met)), and low activity (COMT “AA” (Met/Met) [7]. For example, in healthy individuals, the Val/Val genotype has been linked to improved cognitive performance, particularly in episodic memory and executive function tasks, while the Met/Met genotype has been associated with cognitive decline in response to Tolcapone [6]. In patients with PD, Val/Val carriers were reported to respond more favourably to Entacapone, with prolonged ON time compared to Met/Met carriers [8]. Subpopulation analyses have reported associations between LD response and rs4680 enzyme distribution in various ethnic groups [9,10]. For instance, one study reported an increased susceptibility to LD-induced dyskinesia in Brazilian PD carriers of COMT Met/Met [11], with similar trends observed in Japanese patients with PD [12]. Additionally, genotype differences in COMT variants rs165728 or rs174699 have been associated with variations in LD dosage and susceptibility to dyskinesia in Chinese patients with PD [13].

Furthermore, there is evidence of interethnic variation in the prevalence of rs4680 genotypes. Notably, a higher incidence of Val/Val carriers has been observed among Asian and Black populations compared to Caucasian populations [9,10,14]. However, findings are somewhat conflicting, as other reports suggest a higher frequency of rs4680 in Caucasian cohorts (50%) compared to African (39%) and Oriental (29%) cohorts [15]. Importantly, ethnic-specific differences in PD risk have been observed among Val158Met carriers. For instance, in Japanese patients with PD, the Met/Met genotype has been associated with a higher risk of PD, while the Val/Met genotype appeared protective, a finding that has not been replicated in Caucasian populations [16]. Furthermore, a meta-analysis demonstrated that Val158Met may be associated with PD in the Japanese population, while no such association was found in a Chinese population [17]. Similarly, a more recent meta-analysis suggested that Val158Met may increase PD risk in both Indian and Japanese populations but not in Caucasian groups [18].

Despite the above evidence, interethnic differences in response to COMT- Is are not typically considered in clinical decision-making for PD patient care. It remains uncertain whether this is due to insufficient evidence, methodological barriers in COMT-I studies, or other factors. One possibility is the underrepresentation of ethnic minority groups in clinical trials investigating COMT-I in PD, which has previously been reported [19]. Given that clinical drug trials are considered the gold standard for understanding drug efficacy and dosage, the lack of ethnic diversity in these trials may lead to findings that are influenced by artefacts and reflect responses in selective ethnic groups. To further investigate this issue, we conducted a literature search to assess the inclusion of diversity in published COMT-Is clinical trials.

## 2. Methodology

In this literature review, we performed a narrative analysis, searching for publications on PubMed. To provide a comprehensive overview across different timeframes, we did not limit the search years. The following MeSH terms were used (“Parkinson’s disease”) AND (Opicapone OR Tolcapone OR Entacapone) for trials from inception to 14 April 2024. Inclusion criteria included clinical trial publications investigating COMT-Is in a PD cohort with primary results. The eligibility of publications was assessed by three investigators (NL, SJ, and AR) who screened titles, abstracts, and full-text articles independently from each other. In cases of uncertainty, two senior investigators were consulted to reach a consensus. For each study, the following information was extracted: the type of COMT inhibitor investigated, study design, sample size, and the cohort’s ethnic composition. At the time of the search, there were no review articles addressing the ethnic distribution of COMT-Is trials in PD.

## 3. Results

**Figure 2:**
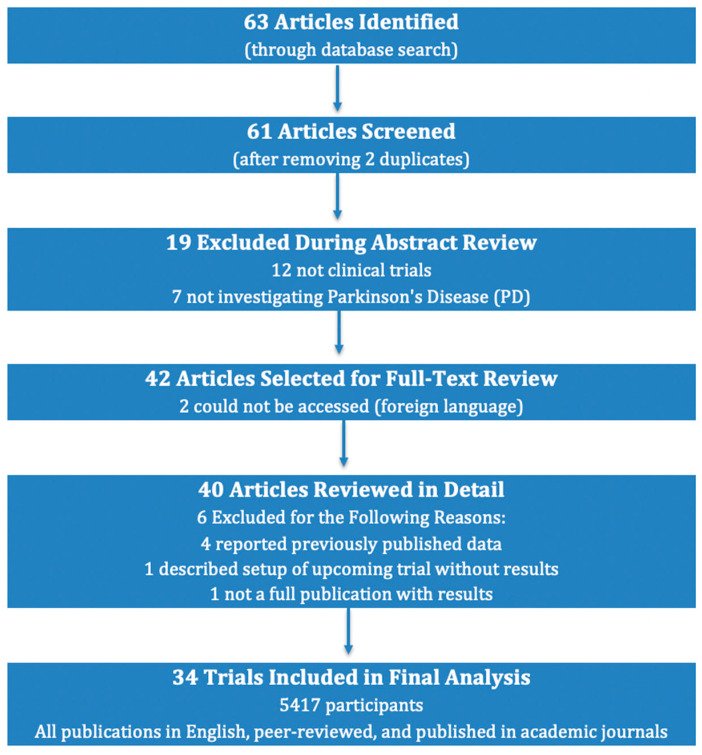
Narrative review article selection procedure.

Our database search identified a total of 63 articles. During the initial identification stage, two duplicates were removed, leaving 61 publications for further review. During the abstract review phase, 19 publications were excluded—12 were not clinical trials, and 7 did not investigate Parkinson’s Disease (PD). This resulted in 42 publications being selected for full-text review; however, two of these could not be accessed as they were in a foreign language. Ultimately, 40 publications were reviewed in detail, with an additional 6 removed for the following reasons: 4 reported previously published data, 1 described the setup of an upcoming trial with no results, and 1 was not a full publication with results. Consequently, a total of 34 trials involving 5417 participants met the study criteria and were included in the final analysis. All included publications were written in English, peer-reviewed, and published in academic journals. Figure 2 provides a detailed overview of the selection process.

### 3.1. Reporting of Ethnicity Data

Many trials that met the inclusion criteria did not report participants’ ethnicities. Among those that did, the data often reflected participants’ nationality or focused predominantly on Caucasian cohorts. Additionally, there were inconsistencies in the reporting of this information—some studies provided details for all enrolled participants, while others only reported on those who were randomized in trial phases. As a result, we documented the available information as it was presented.

### 3.2. Ethnicity Breakdown of All Trials

Of the 34 trials that met the inclusion criteria, 7 investigated Opicapone (2140 participants), 24 investigated Tolcapone (2913 participants), 1 investigated both Tolcapone and Entacapone (14 participants), and 1 investigated both Opicapone and Entacapone (80 participants). Among these, only eight trials provided ethnicity information of participants, including 3 investigating Opicapone, 4 Tolcapone trials, and 1 Opicapone and Entacapone trial (Table 1). Three trials were conducted in Europe, three were conducted worldwide, and two did not specify their location. Both trials investigating Opicapone used a Caucasian-only cohort. Of the four investigating Tolcapone, all specified an individual breakdown of participants’ ethnicities, but showed an over-representation of Caucasian participants. The study investigating Opicapone and Entacapone defined ethnicity according to “white” and “non-white”, with 42 participants constituting the former and 38 the latter. Of the 24 trials that did not specify the ethnicity of participants, 3 investigated Opicapone, 1 investigated Tolcapone and entacapone, and the remaining 20 investigated Tolcapone.

## 4. Discussion

We reviewed published clinical trial cohorts investigating the three main COMT-Is in clinical use for PD (although Tolcapone is no longer in mainstream clinical use), with the aim to clarify and assess the diversity of patients recruited in these studies. Our analysis reveals that out of 34 trials that met our inclusion criteria, 8 reported the ethnic breakdowns of participants. This included three Opicapone studies, four Tolcapone studies and one study investigating both Opicapone and Entacapone. Notably, most of the trials presented a significant overrepresentation of Caucasian subjects, despite the international scope of these trials (Table 1).

While we did not explicitly control for publication time, our findings suggest that this did not correlate with the ethnic composition of trial cohorts or the reporting of participants’ ethnic breakdown. For instance, a clinical trial investigating Tolcapone published in 1996 provided an ethnic breakdown of participants, and despite being overrepresented by Caucasian participants, did include ethnic minority participants [25]. In contrast, two more recent trials on Opicapone conducted in 2015 and 2021, exclusively enrolled Caucasian participants [20,21]. This demonstrates that ethnically homogenous clinical trial cohorts are a persistent, historic problem that remains unresolved. Among all reported COMT-I trials, the BIPARK 2 study, a phase 3 international, randomized, double-blind, placebo-controlled trial investigating Opicapone reported the highest number of ethnic minority participants (102/427) [27]. However, the study did not conduct a sub-analysis of treatment response variability across ethnic groups. The authors did emphasise the need for multicentre studies to enhance diversity in recruitment. Notably, none of the studies reported the inclusion of Black participants, a critical oversight given the variability of COMT enzyme activity in Black individuals with PD and reports of LD hyporesponsiveness in Black patients with PD in the UK [28].

Furthermore, we found that among studies including an ethnic breakdown of clinical trial cohorts, most classified specific non-Caucasian subgroups, except for one study investigating Tolcapone that broadly categorized non-Caucasian participants and did not provide any further specification. Additionally, there were inconsistencies in the reporting of ethnic breakdowns, with some studies noting the ethnicity of all enrolled participants, while others only reported on those who were randomised. This inconsistency makes it challenging to accurately determine the ethnic composition of trial participants, identify at which stage of clinical recruitment the underrepresentation of ethnic groups occurs, and assess the efficacy of COMT inhibitors across different ethnicities.

The above findings are concerning for several reasons. Firstly, COMT is a key regulator of prefrontal dopamine metabolism [29]. It is unsurprising therefore that several studies have reported rs4680 to be associated with cognitive decline. For example, rs4680 has been associated with memory deficits in patients with brain tumours [30] and poorer cognitive performance in individuals with bipolar disorder [31]. This association has been replicated in various ethnic groups, with Met/Met reported to confer a protective effect against cognitive decline in healthy elderly Korean populations [32], while in Mexican populations, Val/Val carriers were found to be at a higher risk of developing dementia [33].

Studies comparing the distribution of the rs4680 COMT variant between ethnic groups are rare, and those that exist often contradict recent findings. For instance, an eight-year study reported that the Val allele exerted a protective effect against cognitive decline in both Black and Caucasian groups, as measured by the Digit Symbol Substitution Test (DSST) [34]. Interestingly, performance on the Modified Mini-Mental State Examination (3MS) was found to be exclusively better among Caucasian Val/Val carriers. Given that these tests measure different cognitive processes, the DSST assessing psychomotor function and the 3MS evaluating various aspects of memory, this suggests that distinct neuropathological processes related to rs4680 may be influencing cognitive outcomes across ethnic groups.

Furthermore, COMT polymorphisms have also been associated with neuropsychiatric treatment response and symptoms, given the significance of rs4680 in schizophrenia and bipolar disorder [35,36,37]. In Chinese Han populations, COMT rs4818 and rs4680 have been associated with susceptibility to treatment-resistant depression [18,38]. More recent reports have attempted to differentiate the association between COMT polymorphisms, pain, and depression in PD cohorts. For example, Met/Met and Val/Met carriers with PD were found to be more susceptible to pain than Val/Val carriers, even when controlling for depression [39]. Additionally, the COMT rs6267 “GT” variant has been associated with an increased susceptibility to pain, a finding supported by previous work that found a higher prevalence of rs6267 carriers with PD-related pain [40]. The underrepresentation of ethnic minority groups in clinical trials further obscures our understanding of COMT genotype-phenotype correlation, preventing clear conclusions. Specifically, this may support clinical decisions regarding COMT-I dosage and prescription, especially given the current challenges in managing non-motor symptoms in patients at advanced stages of PD.

To the best of our knowledge, this is the first review that has investigated the ethnic distribution of participants in clinical trials investigating COMT-Is in PD. However, it is important to acknowledge that barriers to trial participation by minority communities in the West exist across several medical fields. This has been a focal point in the implementation of the King’s model and the Stepped Care strategy for personalised medicine in PD [41,42]. Increasing the diversity of clinical trial cohorts necessitates addressing two primary factors. The first is the barriers faced by ethnic groups themselves and the second is methodological constraints in study design and conduct. As concerns the former, a common misconception is that the under-representation of diverse populations in clinical trials is an extension of their lack of interest [43].

While this review did not investigate patient perspectives on participation, specific factors have repeatedly been found to prevent participation. These include a general sense of mistrust of researchers and the medical system, constraints in time and resources (e.g., taking time off work may be particularly challenging for ethnic minority groups), and a lack of awareness and health literacy. Additionally, language barriers and the absence of relevant information in places regularly frequented by ethnic minority groups further exacerbate this issue [43,44,45]. Another potential factor is that certain comorbidities, which are more prevalent in ethnic minorities and may be exclusion criteria in trials, could also contribute to their under-representation. Liaising with local champions and establishing networks that address barriers at both internal and external levels can enhance understanding of these challenges and ensure that recruitment frameworks are better aligned with the needs of diverse populations [41]

Furthermore, methodological constraints, as identified in this study, often manifest inconsistent reporting of participant demographics. Specifically, we found that numerous publications either omitted ethnicity altogether, categorized participants’ ethnicity based on their country of origin, or utilized vague ethnicity definitions such as ‘other’. This lack of clarity complicates the assessment of trial diversity and hampers the interpretation of results, limiting our ability to accurately evaluate the applicability of findings to diverse populations. There are several strategies that may help to address this. For example, by mandating the disclosure of ethnic distribution in cohorts, researchers will be encouraged to reflect more on the diversity of their clinical trial participants. Establishing clear guidelines for reporting baseline characteristics can mitigate these issues and enhance transparency in clinical trial reporting.

Furthermore, implementing mandatory quotas to recruit ethnic minority groups could effectively increase their representation in clinical trials. For example, a previous study compared two different frameworks for recruiting ethnic minority participants, with one key difference being that one approach encouraged 10% minority participation, while the other mandated 10% inclusion through funding requirements. The study found that the latter approach led to significantly higher participation of ethnic minority groups and contributed to the success of the interventions [46].

Such measures can incentivize researchers and trial sponsors to actively engage with diverse communities, ensuring greater inclusivity in medical research. Additionally, learned societies could offer education and training programs for industry professionals, academics, and clinicians to emphasize the importance of diversity in clinical research and address current challenges. By enhancing awareness and understanding within the industry, these initiatives can catalyse more proactive efforts to incorporate diverse populations in trials.

Importantly, these interventions may also be generalized to other research endeavours beyond clinical trials, helping to streamline clinical care interventions. This includes the integration of technological advancements that have been shown to decrease caregiver burden, improve quality of life, and enhance the efficiency of clinical care. A key example of such technology is the use of wearable devices [47].

## 5. Conclusions

In this narrative review of COMT-I trials and diversity in recruitment, we identified two main findings: First, there is a notable absence of ethnic minority participants, particularly Black participants, in COMT-I trials globally, despite known interethnic differences in COMT enzyme activity. Second, the reporting of ethnicity is inconsistent across studies, with many failing to categorize participants by ethnicity or reporting it at different stages of the clinical trial process. For example, some studies reported ethnicity only at the enrolment stage, while others provided details at the randomisation stage. Failure to address these issues risks perpetuating healthcare disparities and leaves the effects and efficacy of COMT-Is in these populations unclear.

## Figures and Tables

**Table 1:** Summary of COMT inhibitor trials that mentioned the ethnic composition of cohorts. NS: Not stated.

COMT Inhibitor	Location Performed in	Trial Registration	Study Design	Sample Size	Ethnicity		Citation
					Caucasian	Non-Caucasian	
Opicapone	Germany and UK	NCT02847442	Open-label, single-arm, multicentre trial	495	495	0	[20]
Opicapone	Romania (three centres) and Ukraine (four centres).	EudraCT No. 2009-012897-12	Randomized, multicentre, double-blind, placebo-controlled study in four parallel groups	40	40	0	[21]
Tolcapone	92 sites in Europe, the United States, and Canada	NS	Multicentre, randomised, placebo-controlled, double-blind, parallel groups	677	661	3 (Black), 5 (Asian), 6 (Hispanic), 2 (Other)	[22]
Tolcapone	32 centres in Finland, France, Germany, Spain, Sweden, Switzerland, and the United States	NS	Randomized, double-blind, active-controlled study	150	146	4 (Asian)	[23]
Opicapone and Entacapone	Paris, France	EudraCT No. 2011-000173-31	Randomized, double-blind, gender-balanced, placebo-controlled study	80	42	38 (No further specification)	[24]
Tolcapone	NS	NS	Randomized, double-blind	67	59	5 (Indian), 2 (Chinese), 1 (African)	[25]
Tolcapone	NS	NS	Double-blind, randomised, placebo-controlled,	48	47	1 (Oriental)	[26]
Opicapone	Belgium, United Kingdom, Israel, Estonia, Czech Republic, Russia; South Africa, Australia, South Korea, India; Argentina, and Chile	NCT01227655	Randomised, double-blind, placebo-controlled and active-controlled trial	427	294	102 (Asian), 10 (Other)	[27]

## Data Availability

All data presented in this paper are taken from published peer-reviewed papers and are therefore available in the public domain.
